# A novel method for the nondestructive classification of different‐age Citri Reticulatae Pericarpium based on data combination technique

**DOI:** 10.1002/fsn3.2059

**Published:** 2020-12-08

**Authors:** Pao Li, Xinxin Zhang, Yu Zheng, Fei Yang, Liwen Jiang, Xia Liu, Shenghua Ding, Yang Shan

**Affiliations:** ^1^ Hunan Agricultural Product Processing Institute Hunan Academy of Agricultural Sciences Changsha China; ^2^ Hunan Provincial Key Laboratory of Food Science and Biotechnology College of Food Science and Technology Hunan Agricultural University Changsha China; ^3^ School of Medicine Hunan Normal University Changsha China

**Keywords:** chemometric method, Citri Reticulatae Pericarpium, classification, data combination, near‐infrared diffuse reflectance spectroscopy

## Abstract

The quality of Citri Reticulatae Pericarpium (CRP) is closely correlated with the aging time. However, CRPs in different storage ages are similar in appearance, and the young CRP may be labeled as the aged one to obtain the excess profit by some unscrupulous traders. Most traditional analysis methods are laborious and time‐consuming, and they can hardly realize the nondestructive classification. In this paper, a novel method based on near‐infrared diffuse reflectance spectroscopy (NIRDRS) and data combination technique for the nondestructive classification of different‐age CRPs was proposed. The CRPs in different storage ages (5, 10, 15, 20, and 25 years) were measured. The near‐infrared spectra of outer skin and inner capsule were obtained. Principal component analysis (PCA), soft independent modeling of class analogy (SIMCA), and Fisher's linear discriminant analysis (FLD), with different data pretreatment methods, were used for the classification analysis. Data combination of the outer skin and inner capsule spectra was discussed for further improving the classification results. The results show that multiple sensors provide more useful and complementary information than a single sensor does for improving the prediction accuracy. With the help of data combination strategy, 100% prediction accuracy can be obtained with both second‐order derivative–FLD and continuous wavelet transform–multiplicative scatter correction–FLD methods.

## Graphical abstract

A novel method based on near‐infrared diffuse reflectance spectroscopy (NIRDRS) and data combination technique for the nondestructive classification of different‐age CRPs was proposed. With the help of data combination strategy, 100% prediction accuracy can be obtained.

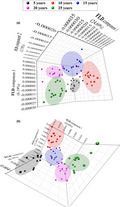

## INTRODUCTION

Citri Reticulatae Pericarpium (CRP), also known as Chenpi in Chinese, is the dried ripe pericarp of *Citrus reticulata* Blanco (Rutaceae). Citri Reticulatae Pericarpium is rich in essential oils, flavonoids, and alkaloids, and can be used to treat indigestion and cardiovascular diseases (Yi et al., 2007). Studies have shown that the quality of CRP is closely correlated with the aging time (Shi et al., 2018). However, CRPs in different storage ages are similar in appearance, and it is difficult to distinguish them for the layman. The young CRP may be labeled as the aged one to obtain the excess profit by some unscrupulous traders. Therefore, it is very important to develop a simple, rapid, and accurate way for the classification of CRPs in different storage ages.

The thickness of peel, the size of secretory cavity, smell, and taste are used as the indicators to distinguish the CRPs in different storage ages. However, it is difficult for consumers and food inspectors due to the similar physical appearance, smell, and taste of CRPs in different storage ages. Instrument analysis is an effective method for the identification analysis, by analyzing the volatile compounds, flavonoids, alkaloids, and phenolic acids in CRPs. Gas chromatography–mass spectrometry (GC‐MS) has been reported to compare comprehensively the volatile constituents in Citri Reticulatae Blanco Pericarpium (CRBP) and Citri Reticulatae Chachi Pericarpium (CRCP), with the help of principal component analysis (PCA) and orthogonal partial least squares discrimination analysis (Duan et al., 2016). The volatile oils and five bioactive flavonoids in CRP collected from different regions were analyzed by GC‐MS and high‐performance liquid chromatography (HPLC) (Luo et al., 2018). Headspace–gas chromatography–ion mobility spectrometry (HS‐GC‐IMS) with PCA method was established to discriminate CRCP and CPBP by their volatile organic compounds (Lv et al., 2020). Ultra‐high‐performance liquid chromatography quadrupole/time‐of‐flight mass spectrometry (UHPLC‐TOF/MS) is an effective method for the differentiation of CRCP and CPBP, and CRCP with different storage ages. 31 metabolites, such as aloesone, roseoside, and 7‐hydroxy‐5,3′,4′‐trimethoxyflavone, were identified to distinguish CRCP in different storage ages (Luo et al., 2019). The results show an upward trend in 3–15 years and a downward trend to a stable state in 15–30 years, indicating that CRCP has the characteristics of “the longer it is stored, the better the quality is.” However, the chromatographic methods usually require expensive equipment and tedious operation. Besides, samples need to be pretreated in these methods and nondestructive testing cannot be realized. Low‐cost, nondestructive, and accurate methods for the classification of CRPs in different storage ages are still demanded.

Near‐infrared diffuse reflectance spectroscopy (NIRDRS) has been widely used in the nondestructive analysis of complex samples in food (Chen et al., 2017; Yu et al., 2009), agriculture (Liu et al., 2016; Purcell et al., 2009; Tardaguila et al., 2017), and medicine industries (Li et al., 2012; Liu et al., 2018; Xu et al., 2015). The information of hydrogen‐containing functional groups such as C‐H, N‐H, S‐H, and O‐H' stretching vibration can be obtained with NIRDRS. However, the useful information of analytes is always embedded in the interference of overlapping and background. A large number of chemometric methods have been developed to solve the problems. Spectral pretreatment methods have been used for the baseline correction and background removal, with different advantages and disadvantages (Bian et al., 2016; Han et al., 2017; Li et al., 2020; Ma, Liu, et al., 2020; Ma, Pang, et al., 2020; Shao et al., 2010). De‐bias correction and detrend (DT) methods can be used to eliminate the interference of baseline drift (Li et al., 2020). Standard normal variate (SNV) transformation and multiplicative scatter correction (MSC) methods are used to eliminate the scattering effect caused by uneven particle distribution and particle size. Maximum and minimum normalization (MinMax) method is a scaling technique that normalizes all the variables into a certain range (Bian et al., 2020). First‐order derivative (1st Der), second‐order derivative (2nd Der), and continuous wavelet transform (CWT) can subtract the influence of instrument background or drift on signal (Bian et al., 2020). However, in the results of higher order derivative, the noise level increases significantly (Li et al., 2019). In addition, single pretreatment method can only suppress one certain interference in the spectra, and the optimal pretreatment method is usually different for different dataset. To solve the problem, the combination pretreatment methods are often used to eliminate various interferences in the spectra (Bian et al., 2020). PCA (Li et al., 2012), soft independent modeling of class analogy (SIMCA) (Szabó et al., 2018), and Fisher's linear discriminant analysis (FLD) (Witjes et al., 2003; Yan et al., 2018) have been applied for the classification, while partial least squares (PLS), boosting partial least squares (Shao et al., 2010), and related robust techniques (Li et al., 2018; Li et al., 2020; Ma, Liu, et al., 2020; Ma, Pang, et al., 2020; Melssen et al., 2007) were used for the quantitative analysis. Multiple sets of data provide more useful and complementary information than a single set. More and more attention was paid to the data combination of near‐ and mid‐infrared spectroscopy, Raman spectroscopy, electronic nose, and electronic tongue. For example, the NIRDRS and HPLC data of lotus seed were combined into a new one to extract more information and submitted for building reliable model (Guo et al., 2017). With the help of data combination method, the quantitative predicting analysis of liensinine, rutin, total sugar, and total polysaccharide in Lotus seed samples can simultaneously and successfully be performed.

Though research about the analysis of complex food samples with the NIRDRS with chemometric methods has been widely reported, the research of identification of CRPs in different storage ages is rare, due to the complexity of the composition of CRP and little differences in the compositions of storage ages (Zhou et al., 2015). The aim of this study was to use NIRDRS instrument and data combination technique to obtain reliable and accurate identification results of CRPs in storage ages. The near‐infrared spectra of outer skin and inner capsule were obtained directly by NIRDRS instrument. PCA, SIMCA, and FLD, with different data pretreatment methods, were used for the classification analysis of CRPs in different storage ages. Data combination of the outer skin and inner capsule spectra was discussed for improving the classification results.

## MATERIALS AND METHODS

### CRP sample

In this study, CRPs in different storage ages (5, 10, 15, 20, and 25 years) were collected from Guangdong Fu Dong Hai Co., Ltd, and 40 samples were taken from each age‐group. Therefore, 200 CPR samples were analyzed.

### Instrumentation and measurements

All spectra were obtained by a MPA spectrometer (Bruker Optics Inc.) in diffuse reflectance mode with integrating sphere diffuse reflection accessory (Bruker Optics Inc.). Each CRP is composed of three petals of pericarp (~50 mm diameter), and a petal for each CRP without destroying was placed directly in the light spot center without the container. Each spectrum is composed of 2,204 data points recorded from 12,000 to 3,500 cm^−1^. The measurements were repeated three times and averaged.

### Data analysis

The Kennard–Stone (KS) method was applied for the partition of the calibration and test set according the 2:1 proportion. 200 samples were divided into a calibration dataset with 133 samples and a test dataset with 67 samples. Besides, to eliminate noise and background interference, the spectra were treated by different pretreatment techniques, such as de‐bias correction, DT, SNV transformation, MinMax, MSC, 1st Der and 2nd Der, CWT, 1st DT, 1st SNV, 1st MSC, and CWT‐SNV and CWT‐MSC to improve the accuracy of classification results. PCA, SIMCA, and FLD methods were used for the classification analysis. The spectra were mean‐centered prior to the creation of the models. For FLD method, the total number of objects should be equal to at least three to five times the number of variables. Therefore, for classification by FLD modeling, PCA was applied to reduce the multidimensionality into fewer principal components (PCs). A maximum of 30 PCs were selected and most variations (~99%) were explained (Wang et al., 2013).

Multiple sets of data may provide more useful and complementary information than a single set. More and more attention was paid to the data combination. Many literatures indicate that better classification results can be obtained when the measured data obtained from different analytical techniques such as near‐ and mid‐infrared spectroscopy, Raman spectroscopy, electronic nose, and electronic tongue were combined (Guo et al., 2017; Zhuang et al., 2014). The appearances and compositions of outer skin and inner capsule are different. Therefore, in this paper, the method of data combination was used to improve the accuracy of classification results in both SIMCA and FLD methods. In the SIMCA method, the matrix of the outer skin (*n* × *m*) and inner capsule spectra (*n* × *m*) were combined into a new matrix (n × 2*m*), and the combination data were obtained. In the FLD method, PCA was applied to the processing of spectral data, which reduced the multidimensionality into fewer PCs. For both outer skin and inner capsule data, most variations (~99%) can be explained with the PC number 30. Therefore, 30 PCs were selected. Scores of these PCs of outer skin and inner capsule were combined as the combination data.

The programs were performed using MATLAB 8.3 ( MathWorks, USA) and run on a personal computer.

## RESULTS AND DISCUSSION

### NIRDRS spectra of CRP samples in different storage ages

**FIGURE 1:**
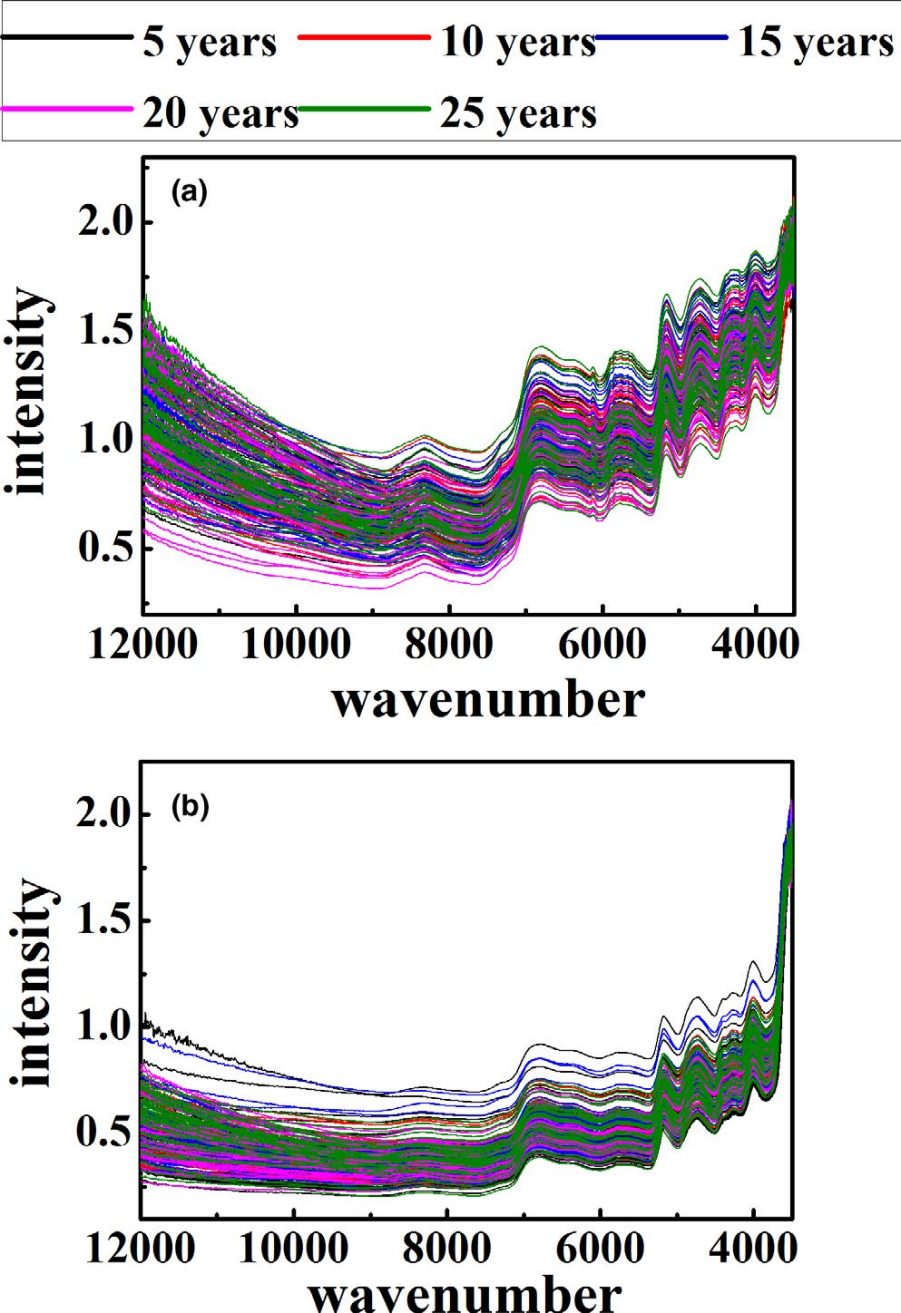
Original spectra of outer skin (a) and inner capsule (b)

Figure 1 shows the original spectra of outer skin and inner capsule. There is very serious background interference in the spectra of both outer skin and inner capsule. The drifting baselines affect the accuracy of the result, due to the unsmooth and rough surface of the CPR sample. Each spectrum has six groups of peaks in the wavenumber range of 9000–8000, 7300–6000, 6000–5500, 5400–4980, 4980–4500, and 4500–4150 cm^−1^, which of peaks belong to the second overtone bands of C‐H, first overtone bands of O‐H, first overtone bands of C‐H, combination bands of O‐H, combination bands of N‐H and O‐H, and combination bands of C‐H, respectively (Guo et al., 2017; Zhou et al., 2015). As shown in the figure, the difference between the inner capsule and outer skin original spectra is very small. Besides, there is almost no difference in the original spectra of CRPs in different storage ages.

### Classification of single spectral data with PCA, SIMCA, and different pretreatment techniques

**FIGURE 2:**
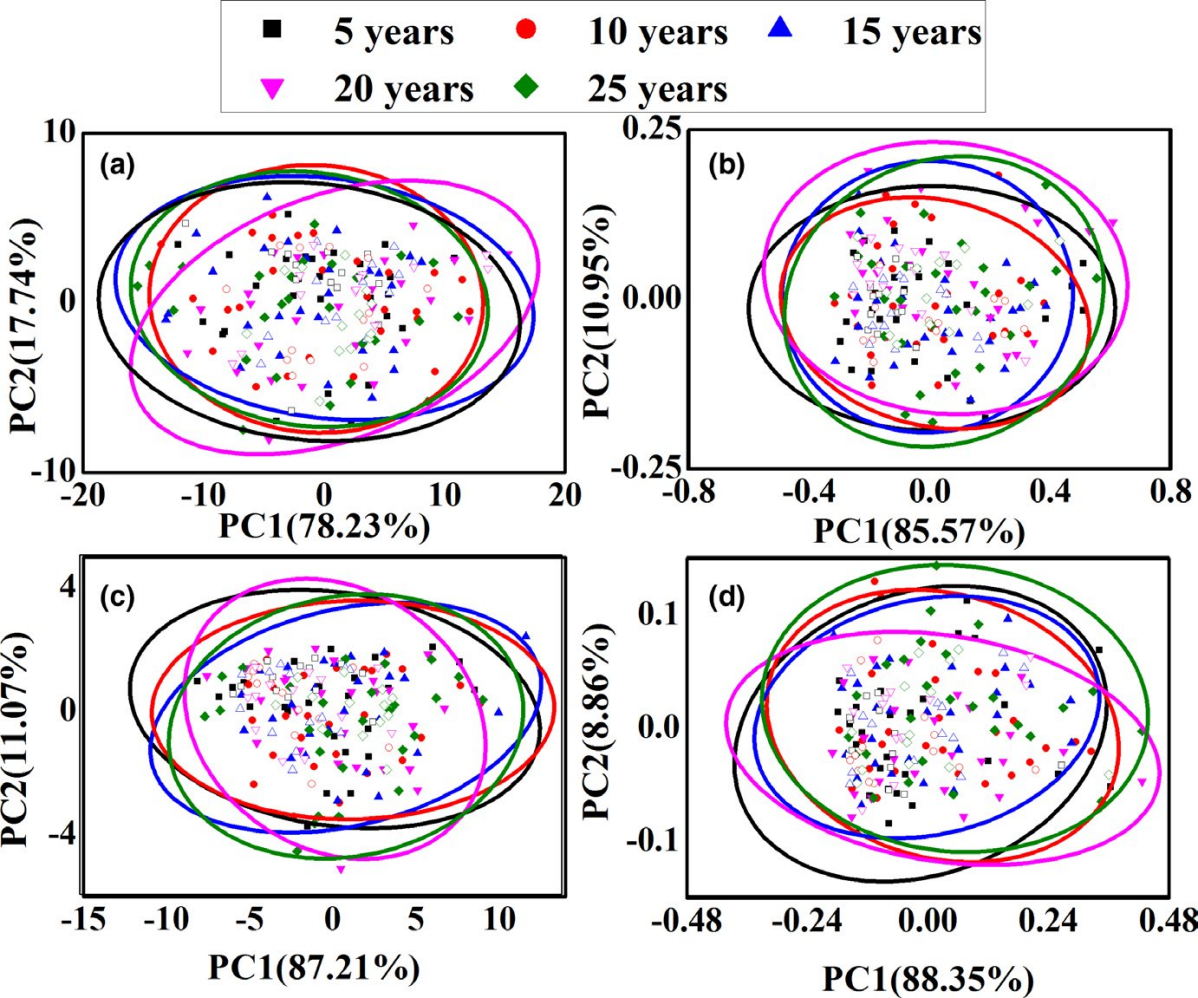
PCA results with different data: raw data (a) and data with MSC pretreatment (b) for the outer skin, and raw data (c) and data with MSC pretreatment (d) for the inner capsule

PCA method was used in the classification analysis. In the calculation, PCA model was established with calibration data, and external verification is carried out with test set. Figure 2a,c shows the PCA results of the outer skin and inner capsule data, respectively. In the figures, the validation sample is marked as void patterns. The first two scores (PC1 and PC2) were used for the classification analysis based on the explanted variances noted in the axis. However, all the groups are merged together. Due to the serious background interference, it may not be feasible to use the raw spectra for the classification analysis. Therefore, different pretreatment techniques were used to eliminate background interference. Figure 2b,d shows the PCA results of the outer skin and inner capsule data with MSC pretreatment, respectively. However, all groups are merged together by MSC method and other pretreatment methods. The results show that it is difficult to get the difference information of CPRs in different storage ages with PCA method.

In order to eliminate background interference and obtain reliable classification models, the classification models were established by SIMCA algorithm with different pretreatment techniques. Table 1 shows the classification accuracies obtained by SIMCA and different pretreatment methods. It is obvious that the classification accuracies with the outer skin spectra are higher than that with the inner capsule spectra. The classification accuracies were improved by using the single pretreatment methods, compared with the results with the raw data. For the analysis of outer skin data, de‐bias and MinMax methods are the best pretreatment methods, and the classification accuracies of whole data are both 79.10%. However, due to the increase of noise level in higher order derivative calculation, the result with 2nd method is not satisfactory. The classification accuracies of 10 and 15 years are 0% with 2nd method. For the analysis of inner capsule data, CWT method is the best pretreatment method, and the classification accuracy of whole data is 74.63%. The classification accuracies of 15 years are the worse for both outer skin and inner capsule data. This is consistent with the change of metabolites in CPRs in different storage ages (Luo et al., 2019). Besides, compared with the results of single pretreatment methods, the classification accuracies were not improved by using the combined pretreatment methods. The useful information is lost with too many pretreatment methods. It seems difficult to perform the classification using SIMCA methods, even when the different pretreatment methods are adopted.

**TABLE 1:** Classification accuracies obtained by SIMCA and different pretreatment methods

Dataset	Pretreatment method	5 years (%)	10 years (%)	15 years (%)	20 years (%)	25 years (%)	Whole data (%)
Outer skin data	Original	61.54	85.71	30.77	100.00	78.57	71.64
	De‐bias	76.92	92.86	46.15	84.62	92.86	79.10
	DT	69.23	85.71	46.15	84.62	78.57	73.13
	SNV	84.62	85.71	38.46	76.92	100.00	77.61
	MinMax	76.92	92.86	30.77	100.00	92.86	79.10
	MSC	76.92	71.43	38.46	84.62	100.00	74.63
	1st	38.46	71.43	15.38	69.23	92.86	58.21
	2nd	7.69	0.00	0.00	53.85	92.86	31.34
	1st DT	30.77	42.86	15.38	61.54	85.71	47.76
	1st SNV	46.15	71.43	7.69	84.62	92.86	61.19
	1st MSC	46.15	71.43	7.69	84.62	92.86	61.19
	CWT	38.46	71.43	7.69	84.62	78.57	56.72
	CWT‐MSC	38.46	71.43	7.69	92.31	78.57	58.21
	CWT‐SNV	38.46	71.43	7.69	92.31	71.43	56.72
Inner capsule data	Original	46.15	92.86	38.46	46.15	85.71	62.69
	De‐bias	30.77	85.71	30.77	53.85	92.86	59.70
	DT	46.15	85.71	38.46	53.85	85.71	62.69
	SNV	38.46	78.57	46.15	69.23	100.00	67.16
	MinMax	61.54	85.71	38.46	69.23	92.86	70.15
	MSC	38.46	78.57	38.46	61.54	100.00	64.18
	1st	23.08	78.57	53.85	53.85	85.71	59.70
	2nd	7.69	64.29	15.38	69.23	71.43	46.27
	1st DT	15.38	71.43	46.15	46.15	100.00	56.72
	1st SNV	38.46	71.43	46.15	69.23	100.00	65.67
	1st MSC	38.46	71.43	46.15	69.23	100.00	65.67
	CWT	53.85	85.71	61.54	76.92	92.86	74.63
	CWT‐MSC	46.15	92.86	46.15	76.92	92.86	71.64
	CWT‐SNV	46.15	92.86	46.15	84.62	92.86	73.13

### Classification of single spectral data with FLD and different pretreatment techniques

FLD method is a powerful supervised classification method, which is used to find the optimal boundary between object classes. Therefore, in the study, FLD method was used for the classification analysis of CRP samples. The results of inner capsule and outer skin data were compared. Different pretreatment techniques were applied to further optimize the classification model. Table 2 shows the classification accuracies with FLD and different pretreatment methods. It is obvious that the classification accuracies of FLD method are higher than that of SIMCA method. For FLD method, the accuracies of the outer skin spectra with different pretreatment methods are more than 88.06%, while the accuracies of the inner capsule spectra are more than 91.04%. For the analysis of outer skin data, de‐bias is the best pretreatment method and the classification accuracies of the whole data are 94.03%. For the analysis of inner capsule data, CWT method is the best pretreatment method and the classification accuracy of the whole data is 98.51%.

**TABLE 2:** Classification accuracies obtained by FLD and different pretreatment methods

Dataset	Pretreatment method	5 years (%)	10 years (%)	15 years (%)	20 years (%)	25 years (%)	Whole data (%)
Outer skin data	Original	92.31	92.86	76.92	92.31	100	91.04
	De‐bias	92.31	92.86	84.62	100	100	94.03
	DT	92.31	92.86	76.92	92.31	100	91.04
	SNV	92.31	92.86	76.92	92.31	100	91.04
	MinMax	100	92.86	76.92	100	100	94.03
	MSC	92.31	92.86	76.92	100	100	92.54
	1st	92.31	92.86	69.23	92.31	100	89.55
	2nd	92.31	92.86	76.92	76.92	92.86	86.57
	1st DT	92.31	92.86	69.23	84.62	100	88.06
	1st SNV	92.31	92.86	69.23	84.62	100	88.06
	1st MSC	92.31	92.86	69.23	76.92	100	86.57
	CWT	92.31	92.86	69.23	92.31	100	89.55
	CWT‐MSC	92.31	92.86	69.23	84.62	100	88.06
	CWT‐SNV	92.31	92.86	69.23	84.62	100	88.06
Inner capsule data	Original	92.31	100	84.62	84.62	92.86	91.04
	De‐bias	100	100	84.62	76.92	92.86	91.04
	DT	92.31	100	84.62	84.62	100	92.54
	SNV	92.31	100	84.62	92.31	92.86	92.54
	MinMax	100	100	84.62	84.62	92.86	92.54
	MSC	92.31	100	84.62	84.62	92.86	91.04
	1st	92.31	100	92.31	92.31	92.86	94.03
	2nd	92.31	100	76.92	92.31	100	92.54
	1st DT	100	100	92.31	100	100	98.51
	1st SNV	84.62	100	92.31	100	92.86	94.03
	1st MSC	92.31	100	92.31	100	100	97.01
	CWT	92.31	100	92.31	92.31	100	95.52
	CWT‐MSC	84.62	100	92.31	100	100	95.52
	CWT‐SNV	92.31	100	92.31	100	100	97.01

### Classification of the combination data with SIMCA and different pretreatment techniques

Multiple sensors may provide more useful and complementary information than a single sensor does for improving the prediction results (Guo et al., 2017; Zhuang et al., 2014). Therefore, data combination method was developed to improve the accuracy of classification results in SIMCA method. Different pretreatment techniques were used to optimize the classification model. Table 3 shows the classification accuracies of the combination data with SIMCA and different pretreatment methods. Compared with the results of single outer skin and inner capsule spectra, the classification accuracies with SIMCA method have been further improved. The accuracies of identification are more than 80% with de‐bias, SNV, and MinMax methods. De‐bias is the best pretreatment method, and the classification accuracy for the whole data is 85.07%. However, the results are still unsatisfactory, especially for the identification of 15‐year CRP.

**TABLE 3:** Classification accuracies obtained by the combination data with SIMCA and FLD methods

Method	Pretreatment method	5 years (%)	10 years (%)	15 years (%)	20 years (%)	25 years (%)	Whole data (%)
SIMCA	Original	53.85	71.43	38.46	100.00	78.57	68.66
	De‐bias	92.31	85.71	69.23	100.00	78.57	85.07
	DT	69.23	78.57	38.46	92.31	78.57	71.64
	SNV	100.00	78.57	53.85	92.31	92.86	83.58
	MinMax	84.62	78.57	53.85	100.00	92.86	82.09
	MSC	61.54	71.43	46.15	100.00	92.86	74.63
	1st	38.46	78.57	15.38	100.00	92.86	65.67
	2nd	0.00	7.14	0.00	61.54	78.57	29.85
	1st DT	23.08	57.14	15.38	84.62	85.71	53.73
	1st SNV	46.15	71.43	15.38	100.00	85.71	64.18
	1st MSC	46.15	85.71	15.38	92.31	85.71	65.67
	CWT	53.85	71.43	15.38	100.00	85.71	65.67
	CWT‐MSC	53.85	64.29	15.38	92.31	85.71	62.69
	CWT‐SNV	53.85	64.29	15.38	92.31	78.57	61.19
FLD	Original	100	92.86	92.31	84.62	100	94.03
	De‐bias	100	100	92.31	92.31	100	97.01
	DT	100	100	84.62	92.31	100	95.52
	SNV	100	100	92.31	84.62	100	95.52
	MinMax	100	100	92.31	92.31	100	97.01
	MSC	100	100	92.31	84.62	100	95.52
	1st	100	92.86	92.31	100	100	97.01
	2nd	100	100	100	100	100	100
	1st DT	100	100	92.31	92.31	92.86	95.52
	1st SNV	100	100	92.31	100	100	98.51
	1st MSC	100	100	84.62	100	100	97.01
	CWT	100	92.86	92.31	100	100	97.01
	CWT‐MSC	100	100	100	100	100	100
	CWT‐SNV	100	100	84.62	100	100	97.01

### Classification of the combination data with FLD and different pretreatment techniques

**FIGURE 3:**
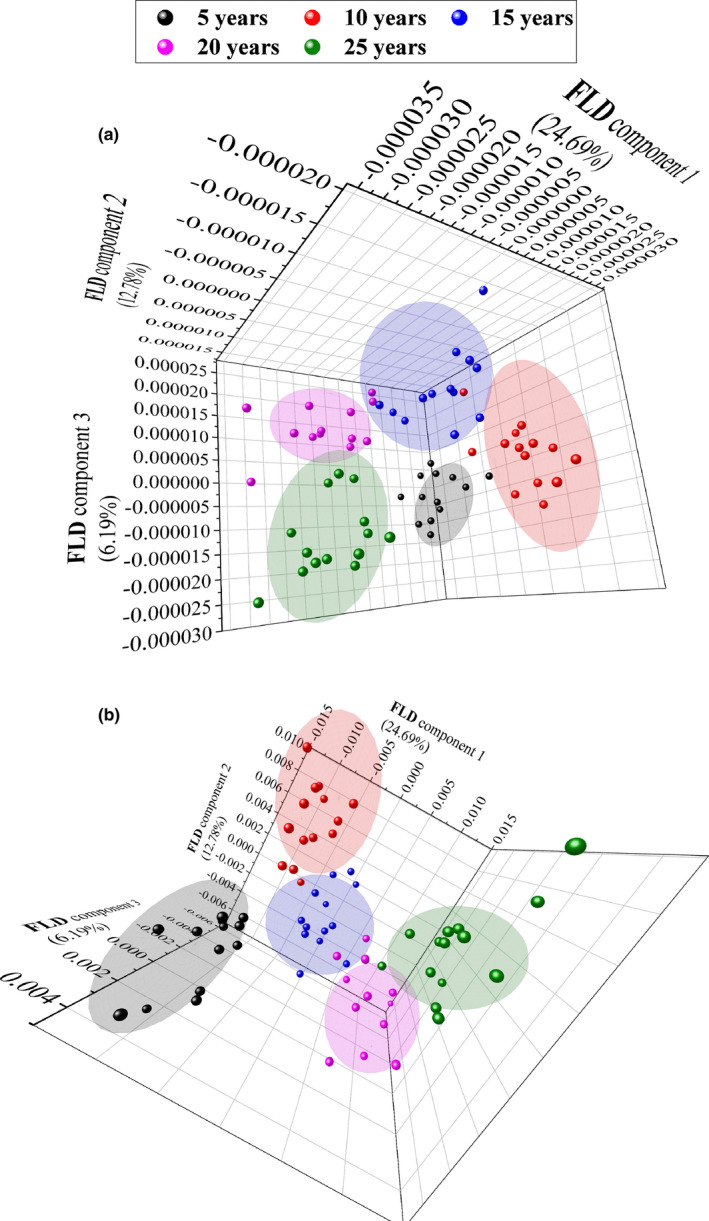
FLD score plot for discrimination of CRPs in different storage ages with second‐order derivative–FLD (a) and CWT‐MSC‐FLD methods (b)

Data combination with FLD and different pretreatment techniques were used to get accurate classification results. Table 3 shows the classification accuracies of the combination data obtained by FLD and different pretreatment methods. The classification accuracies with FLD method are further improved with the combination data, compared with the results of outer skin and inner capsule spectra. The identification accuracy of combination data is more than 94.03% even without spectral pretreatment, while the accuracy of identification is 91.04% with the single spectral data. Spectral pretreatment methods can further optimize the classification model. Data combination models based on second‐order derivative–FLD and continuous wavelet transform–multiplicative scatter correction–FLD obtained best results with 100% prediction accuracy. Furthermore, Figure 3 is the FLD score plot for discrimination of CRPs in different storage ages with second‐order derivative–FLD and CWT‐MSC‐FLD methods, and all the five groups were visually separated. The results demonstrate that the classification of CRP in different storage ages can be achieved by data combination and appropriate chemometric methods.

## CONCLUSION

A simple and nondestructive method for the classification of CRPs in different storage ages was established by using NIRDRS combined with appropriate chemometric methods. Data pretreatment methods can eliminate the background interference, and data combination method can significantly improve the accuracy of classification. Data combination models based on second‐order derivative–FLD and CWT‐MSC‐FLD obtained best results with 100% prediction accuracy. The developed technology based on data combination and appropriate chemometric methods can be regarded as a fast, nondestructive, and accurate way for the classification of CRPs in different storage ages and has vast application prospect.

## CONFLICT OF INTEREST

The authors declared that they have no conflicts of interest to this work.

## References

[fsn32059-bib-0001] Bian, X. H. , Li, S. J. , Shao, X. G. , & Liu, P. (2016). Variable space boosting partial least squares for multivariate calibration of near‐infrared spectroscopy. Chemometrics and Intelligent Laboratory Systems, 158, 174–179. 10.1016/j.chemolab.2016.08.005

[fsn32059-bib-0002] Bian, X. H. , Wang, K. Y. , Tan, E. X. , Diwu, P. Y. , Zhang, F. , & Guo, Y. G. (2020). A selective ensemble preprocessing strategy for near‐infrared spectral quantitative analysis of complex samples. Chemometrics and Intelligent Laboratory Systems, 197, 103916 10.1016/j.chemolab.2019.103916

[fsn32059-bib-0003] Chen, J. , Zhu, S. , & Zhao, G. (2017). Rapid determination of total protein and wet gluten in commercial wheat flour using siSVR‐NIR. Food Chemistry, 221, 1939–1946. 10.1016/j.foodchem.2016.11.155 27979183

[fsn32059-bib-0004] Duan, L. I. , Guo, L. , Dou, L.‐L. , Zhou, C.‐L. , Xu, F.‐G. , Zheng, G.‐D. , Li, P. , & Liu, E.‐H. (2016). Discrimination of Citrus reticulata Blanco and Citrus reticulata ‘Chachi’ by gas chromatograph‐mass spectrometry based metabolomics approach. Food Chemistry, 212, 123–127. 10.1016/j.foodchem.2016.05.141 27374515

[fsn32059-bib-0005] Guo, Y. , Ding, X. X. , & Ni, Y. N. (2017). The combination of NIR spectroscopy and HPLC chromatography for differentiating lotus seed cultivars and quantitative prediction of four main constituents in lotus with the aid of chemometrics. Analytical Methods, 9(45), 6420–6429. 10.1039/C7AY02021J

[fsn32059-bib-0006] Han, X. , Huang, Z. X. , Chen, X. D. , Li, Q. F. , Xu, K. X. , & Chen, D. (2017). On‐line multi‐component analysis of gases for mud logging industry using data driven Raman spectroscopy. Fuel, 207, 146–153. 10.1016/j.fuel.2017.06.045

[fsn32059-bib-0007] Li, P. , Du, G. R. , Cai, W. S. , & Shao, X. G. (2012). Rapid and nondestructive analysis of pharmaceutical products using near‐infrared diffuse reflectance spectroscopy. Journal of Pharmaceutical and Biomedical Analysis, 70(21), 288–294. 10.1016/j.jpba.2012.07.013 22858521

[fsn32059-bib-0008] Li, P. , Du, G. R. , Ma, Y. J. , Zhou, J. , & Jiang, L. W. (2018). A novel multivariate calibration method based on variable adaptive boosting partial least squares algorithm. Chemometrics and Intelligent Laboratory Systems, 176, 157–161. 10.1016/j.chemolab.2018.03.013

[fsn32059-bib-0009] Li, P. , Li, S. K. , Du, G. R. , Jiang, L. W. , Liu, X. , Ding, S. H. , & Shan, Y. (2020). A simple and nondestructive approach for the analysis of soluble solid content in citrus by using portable visible to near‐infrared spectroscopy. Food Science & Nutrition, 8(5), 2543–2552. 10.1002/fsn3.1550 32405410 PMC7215219

[fsn32059-bib-0010] Li, P. , Shen, R. J. , Li, S. K. , Shan, Y. , Ding, S. H. , Jiang, L. W. , Xia, L. , & Du, G. R. (2019). Nondestructive identification of green tea based on near infrared spectroscopy and chemometric methods. Spectroscopy and Spectral Analysis, 39, 2584–2589. 10.3964/j.issn.1000-0593(2019)08-2584-06

[fsn32059-bib-0011] Liu, P. , Wang, J. , Li, Q. , Gao, J. , Tan, X. , & Bian, X. (2018). Rapid identification and quantification of Panax notoginseng with its adulterants by near infrared spectroscopy combined with chemometrics. Spectrochimica Acta Part A: Molecular and Biomolecular Spectroscopy, 206, 23–30. 10.1016/j.saa.2018.07.094 30077893

[fsn32059-bib-0012] Liu, Y. , Cai, W. S. , & Shao, X. G. (2016). Linear model correction: A method for transferring a near‐infrared multivariate calibration model without standard samples. Spectrochimica Acta Part A: Molecular and Biomolecular Spectroscopy, 169, 197–201. 10.1016/j.saa.2016.06.041 27380302

[fsn32059-bib-0013] Luo, M. X. , Luo, H. J. , Hu, P. J. , Yang, Y. T. , Wu, B. , & Zheng, G. D. (2018). Evaluation of chemical components in Citri Reticulatae Pericarpium of different cultivars collected from different regions by GC–MS and HPLC. Food Science & Nutrition, 6(2), 400–416. 10.1002/fsn3.569 29564108 PMC5849905

[fsn32059-bib-0014] Luo, Y. , Zeng, W. , Huang, K. E. , Li, D. X. , Chen, W. , Yu, X. Q. , & Ke, X. H. (2019). Discrimination of Citrus reticulata Blanco and Citrus reticulata ‘Chachi’ as well as the Citrus reticulata ‘Chachi’ within different storage years using ultra high performance liquid chromatography quadrupole/time‐of‐flight mass spectrometry based metabolomics approach. Journal of Pharmaceutical and Biomedical Analysis, 171, 218–231. 10.1016/j.jpba.2019.03.056 31072532

[fsn32059-bib-0015] Lv, W. , Lin, T. , Ren, Z. , Jiang, Y. , Zhang, J. , Bi, F. , Gu, L. , Hou, H. , & He, J. (2020). Rapid discrimination of Citrus reticulata ‘Chachi’ by headspace‐gas chromatography‐ion mobility spectrometry fingerprints combined with principal component analysis. Food Research International, 131, 108985 10.1016/j.foodres.2020.108985 32247443

[fsn32059-bib-0016] Ma, L. , Liu, D. , Du, C. , Lin, L. , Zhu, J. , Huang, X. , Liao, Y. , & Wu, Z. (2020). Novel NIR modeling design and assignment in process quality control of Honeysuckle flower by QbD. Spectrochimica Acta Part A: Molecular and Biomolecular Spectroscopy, 242, 118740 10.1016/j.saa.2020.118740 PMC7369169 32736221

[fsn32059-bib-0017] Ma, X. P. , Pang, J. F. , Dong, R. N. , Tang, C. , Shu, Y. X. , & Li, Y. K. (2020). Rapid prediction of multiple wine quality parameters using infrared spectroscopy coupling with chemometric methods. Journal of Food Composition and Analysis, 91, 103509 10.1016/j.jfca.2020.103509

[fsn32059-bib-0018] Melssen, W. , Üstün, B. , & Buydens, L. (2007). SOMPLS: A supervised self‐organising map–partial least squares algorithm for multivariate regression problems. Chemometrics and Intelligent Laboratory Systems, 86(1), 102–120. 10.1016/j.chemolab.2006.08.013

[fsn32059-bib-0019] Purcell, D. E. , O'Shea, M. G. , Johnson, R. A. , & Kokot, S. (2009). Near‐infrared spectroscopy for the prediction of disease ratings for Fiji leaf gall in sugarcane clones. Applied Spectroscopy, 63(4), 450–457. 10.1366/000370209787944370 19366512

[fsn32059-bib-0020] Shao, X. G. , Bian, X. H. , & Cai, W. S. (2010). An improved boosting partial least squares method for near‐infrared spectroscopic quantitative analysis. Analytica Chimica Acta, 666(1), 32–37. 10.1016/j.aca.2010.03.036 20433961

[fsn32059-bib-0021] Shi, Q. R. , Guo, T. T. , Yin, T. J. , Wang, Z. Q. , Li, C. H. , Sun, X. , & Yuan, W. H. (2018). Classification of Pericarpium Citri reticulatae of different ages by using a voltammetric electronic tongue system. International Journal of Electrochemical Science, 13, 11359–11374. 10.20964/2018.12.45

[fsn32059-bib-0022] Szabó, É. , Gergely, S. , & Salgó, A. (2018). Linear discriminant analysis, partial least squares discriminant analysis, and soft independent modeling of class analogy of experimental and simulated near‐infrared spectra of a cultivation medium for mammalian cells. Journal of Chemometrics, 32(4), e3005 10.1002/cem.3005

[fsn32059-bib-0023] Tardaguila, J. , Fernández‐Novales, J. , Gutiérrez, S. , & Paz Diago, M. (2017). Non‐destructive assessment of grapevine water status in the field using a portable NIR spectrophotometer. Journal of the Science of Food and Agriculture, 97, 3772–3780. 10.1002/jsfa.8241 28133743

[fsn32059-bib-0024] Wang, S. Q. , Li, W. D. , Li, J. , & Liu, X. S. (2013). Prediction of soil texture using FT‐NIR spectroscopy and PXRF spectrometry with data fusion. Soil Science, 178(11), 626–638. 10.1097/SS.0000000000000026

[fsn32059-bib-0025] Witjes, H. , Rijpkema, M. , van der Graaf, M. , Melssen, W. , Heerschap, A. , & Buydens, L. (2003). Multispectral magnetic resonance image analysis using principal component and linear discriminant analysis. Journal of Magnetic Resonance Imaging, 17(2), 261–269. 10.1002/jmri.10237 12541234

[fsn32059-bib-0026] Xu, Z. H. , Liu, Y. , Li, X. Y. , Cai, W. S. , & Shao, X. G. (2015). Discriminant analysis of Chinese patent medicines based on near‐infrared spectroscopy and principal component discriminant transformation. Spectrochimica Acta Part A: Molecular and Biomolecular Spectroscopy, 149, 985–990. 10.1016/j.saa.2015.05.030 26010567

[fsn32059-bib-0027] Yan, S. , Lai, X. X. , Du, G. R. , & Xiang, Y. H. (2018). Identification of aminoglycoside antibiotics in milk matrix with a colorimetric sensor array and pattern recognition methods. Analytica Chimica Acta, 1034, 153–160. 10.1016/j.aca.2018.06.004 30193629

[fsn32059-bib-0028] Yi, L. Z. , Yuan, D. L. , Liang, Y. Z. , Xie, P. S. , & Zhao, Y. (2007). Quality control and discrimination of Pericarpium Citri Reticulatae and Pericarpium Citri Reticulatae Viride based on high‐performance liquid chromatographic fingerprints and multivariate statistical analysis. Analytica Chimica Acta, 588(2), 207–215. 10.1016/j.aca.2007.02.012 17386812

[fsn32059-bib-0029] Yu, H. Y. , Niu, X. Y. , Lin, H. J. , Ying, Y. B. , Li, B. B. , & Pan, X. X. (2009). A feasibility study on on‐line determination of rice wine composition by Vis–NIR spectroscopy and least‐squares support vector machines. Food Chemistry, 113(1), 291–296. 10.1016/j.foodchem.2008.06.083

[fsn32059-bib-0030] Zhou, Z. , Li, Y. , Zhang, Q. , Shi, X. Y. , Wu, Z. S. , & Qiao, Y. J. (2015). Comparison of ensemble strategies in online NIR for Monitoring the extraction process of Pericarpium citri reticulatae based on different variable selections. Planta Medica, 82(01/02), 154–162. 10.1055/s-0035-1558085 26485639

[fsn32059-bib-0031] Zhuang, H. , Ni, Y. N. , & Kokot, S. (2014). Combining HPLC–DAD and ICP‐MS data for improved analysis of complex samples: Classification of the root samples from Cortex moutan. Chemometrics and Intelligent Laboratory Systems, 135, 183–191. 10.1016/j.chemolab.2014.04.018

